# The effect of mere measurement from a cardiovascular examination program on physical activity and sedentary time in an adult population

**DOI:** 10.1186/s13102-018-0090-8

**Published:** 2018-01-23

**Authors:** Lisa Voigt, Sophie Baumann, Antje Ullrich, Franziska Weymar, Ulrich John, Sabina Ulbricht

**Affiliations:** 1 Institute of Social Medicine and Prevention, University Medicine Greifswald, Greifswald, Walther-Rathenau-Str. 48, D-17475 Greifswald, Germany; 2 Partner site Greifswald, German Centre for Cardiovascular Research (DZHK), Fleischmannstr. 42-44, D-17475 Greifswald, Germany; 3 Institute for Community Medicine, Section Epidemiology of Health Care and Community Health, University Medicine Greifswald, Ellernholzstr. 1-2, D-17487 Greifswald, Germany

**Keywords:** Question-behavior effect, Measurement reactivity, Research participation, Brief intervention, Random-effects modelling

## Abstract

**Background:**

Measuring physical activity (PA) and sedentary time (ST) by self-report or device as well as assessing related health factors may alter those behaviors. Thus, in intervention trials assessments may bias intervention effects. The aim of our study was to examine whether leisure-time PA, transport-related PA, and overall ST measured via self-report vary after assessments and whether a brief tailored letter intervention has an additional effect.

**Methods:**

Among a sample of subjects with no history of myocardial infarction, stroke, or vascular intervention, a number of 175 individuals participated in a study comprising multiple repeated assessments. Of those, 153 were analyzed (mean age 54.5 years, standard deviation = 6.2; 64% women). At baseline, participants attended a cardiovascular examination (standardized measurement of blood pressure and waist circumference, blood sample taking) and wore an accelerometer for seven days. At baseline and after 1, 6, and 12 months, participants completed the International Physical Activity Questionnaire. A random subsample received a tailored counseling letter intervention at month 1, 3, and 4. Changes in PA and ST from baseline to 12-month follow-up were analyzed using random-effects modelling.

**Results:**

From baseline to 1-month assessment, leisure-time PA did not change (Incidence rate ratio = 1.13, *p* = .432), transport-related PA increased (Incidence rate ratio = 1.45, *p* = .023), and overall ST tended to decrease (b = − 1.96, *p* = .060). Further, overall ST decreased from month 6 to month 12 (b = − 0.52, *p* = .037). Time trends of the intervention group did not differ significantly from those of the assessment-only group.

**Conclusions:**

Results suggest an effect of measurements on PA and ST. Data of random-effects modelling results revealed an increase of transport-related PA after baseline to 1-month assessment. Decreases in overall ST may result from repeated assessments. A brief tailored letter intervention seemed to have no additional effect. Thus, measurement effects should be considered when planning intervention studies and interpreting intervention effects.

**Trial registration:**

ClinicalTrials.gov NCT02990039. Registered 7 December 2016. Retrospectively registered.

## Background

Assessments are essential in order to determine the initial level of outcome-related variables, to monitor the progress over the course of the study, and to collect the outcome measures [1]. In trials aimed to increase physical activity (PA) or to reduce sedentary time (ST), measurements may comprise (i) self-reported frequency and duration of PA and ST as well as related cognitions, (ii) objectively measured PA and ST using technical devices, e.g., accelerometer, and (iii) physical examinations, e.g., standardized measurement of blood pressure or waist circumference. However, assessing past behavior, intentions, or other related cognitions may change the behavior that is investigated. This phenomenon is known as mere-measurement effect (MME) [2]. Altering behavior as a result of MME may occur because (i) attitudes towards the behavior are more accessible, (ii) cognitive dissonance is raised when realizing a desirable behavior is not performed, or (iii) the behavior is simulated in the mind which increases likelihood of performance at the next opportunity [3, 4]. If participants of intervention trials change their behavior as a reaction to baseline assessments this may introduce bias to the investigated intervention outcomes [2, 5, 6]. Both participants in the intervention and in the control group may alter their behavior in a way similar to the behavior change that is intended by an intervention, thus, intervention effects may be underestimated. It was also suggested that baseline assessments may increase receptiveness to an intervention. This could yield some kind of synergetic effect which may result in an overestimation of intervention effects [7, 8].

There is evidence that measuring PA by self-report or device as well as measuring related constructs changes various PA outcomes assessed by self-report [9, 10] or device [11, 12]. Two recent meta-analyses [4, 13] found small effect sizes for MME, nevertheless, both suggested that estimates were inflated due to publication bias. Moreover, it was found that several studies showed considerable risk of bias indicating further overestimation of the small effect size [13, 14]. Thus, evidence on MME remains inconclusive.

Although MME poses a problem in intervention trials [2], researchers usually do not examine whether changes in the target behavior occurred under absence of any intervention components, that is, due to MME. Further, studies investigating MME mostly assess outcomes after a short period of time, for example, 6 weeks [15–17] without an extended follow-up. Finally, we are not aware of studies investigating MME on ST.

The aim of our study was (i) to identify potential MME of a cardiovascular examination program on PA and ST indicated by significant differences in leisure-time PA, transport-related PA, and overall ST between baseline assessment and 12-month follow-up measured via self-report in a sample of apparently healthy adults and (ii) to investigate whether a brief tailored letter intervention may have an additional effect indicated by differences over time in a respective subsample.

## Methods

### Study sample

**Fig. 1:**
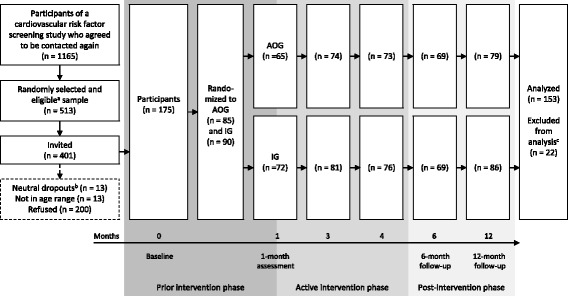
Flow of participation and study design. AOG = Assessment-only group. IG = Intervention group. Assessments at baseline and at 12 months included: paper-pencil questionnaires on socio-demographics, physical activity, and sedentary time as well as physical examination (standardized measurement of blood pressure, body height, body weight, and waist- and hip-circumference, blood sample taking) and 7-day-accelerometry. Assessments at months 1, 3, 4, and 6 included: paper-pencil questionnaires on physical activity and sedentary time. ^a^ Eligibility criteria: age ≥ 40 and ≤ 65 years, no history of cardiovascular event (myocardial infarction or stroke) or vascular intervention, self-reported body mass index ≤ 35 kg/m^2^, resident in a pre-defined zip-code area. ^b^ had died, had a cardiovascular event or intervention, were too ill to participate, or moved away. ^c^ due to late response

As described elsewhere [18], persons aged between 40 and 75 years were recruited for a prior study between June 2012 and December 2013 in general practices, job centers, and via one statutory health insurance. A random sample of 513 people was drawn from individuals who agreed to be contacted again (*n* = 1165, 95%) and fulfilled the following eligibility criteria: age between 40 and 65 years, no history of cardiovascular event (myocardial infarction or stroke) or vascular intervention, self-reported body mass index ≤ 35 kg/m^2^, and resident in a pre-defined zip-code area. Among them, 401 persons were contacted and invited to participate in a study aimed to assess the feasibility of a tailored counselling letter intervention to increase PA and to reduce ST during leisure time. A number of 175 agreed to participate and gave written informed consent. For analyses, 22 cases were excluded because they severely exceeded the given time frame of 2 weeks to respond to assessments. Thus, the final sample comprised 153 individuals (Fig. 1).

### Procedure

The current study was conducted between February 2015 and August 2016. All participants were invited to the cardiovascular examination center of the University Medicine Greifswald, where they received blood sample taking and standardized measurement of blood pressure, waist circumference, body height, and body weight. Afterwards, they wore an accelerometer for 7 days. Prior to the examination, participants completed a paper-pencil questionnaire on PA and ST. After baseline assessments, participants were randomized into an assessment-only group (*n* = 85) and an intervention group (*n* = 90). Self-administered assessments regarding PA and ST were conducted at month 1, 3, 4, 6, and 12. In addition, at 12-month follow-up, participants underwent the same procedure and assessments as at baseline. Only individuals of the intervention group received up to three tailored letters to their self-reported PA and ST at month 1, 3, and 4 (Fig. 1).

The study was approved by the clinical ethical committee of the University Medicine Greifswald (protocol number BB 002/15a).

### Measures

#### Physical activity and sedentary time

To assess PA and ST at baseline and at month 1, 6, and 12, the International Physical Activity Questionnaire (IPAQ) was used [19]. The IPAQ measures frequency, duration, and intensity of PA during the last 7 days in various domains of life as well as overall time spent sedentarily during weekdays and weekends. The leisure-time domain includes walking, PA on a moderate-intensity level, and PA on a vigorous-intensity level. The transportation domain includes walking and cycling. In order to sum time spent in PA within one domain, amounts of time spent in one activity are multiplied by their metabolic equivalent of task (MET) values which account for the intensity of the activity. Leisure-time and transport-related PA in MET-hours per week and overall ST in hours per week were calculated according to the IPAQ protocol [20].

#### Socio-demographic, health, and context variables

Several socio-demographic, health, and context variables were collected and considered as covariates for analyses: Socio-demographics were obtained by a self-administrative questionnaire at baseline including sex, age (in years), educational level (< 10, 10, > 10 years), employment status (full-time or part-time, irregularly, not employed), and current partnership (yes, no). Body mass index (< 25 kg/m^2^, ≥ 25 kg/m^2^ and < 30 kg/m^2^, ≥ 30 kg/m^2^) was calculated from body height (using digital scales MZ 10020, ADE GmbH & Co., Hamburg, Germany) and weight (using digital scales SOEHNLE 7720, Soehnle Industrial Solutions GmbH, Backnang, Germany). Context variables included season at baseline data collection (winter, spring, summer) and setting of initial recruitment (general practices, job centers, statutory health insurance).

### Statistical analyses

Latent growth models [21] were used to examine changes in leisure-time PA, transport-related PA, and overall ST over a period of 12 months. MME was indicated by significant differences of those outcomes between baseline and 1-month assessment, that is, before the intervention started and by significant changes between month 1 and 12 in the assessment-only group. *P*-values below .05 were considered statistically significant. Using latent growth models enables to model complex non-linear outcome growth curves, to capture individual differences, and to properly estimate models with missing data [22]. To account for non-linear associations between the outcomes and time, a piecewise model approach was used. Thus, time was divided into intervals at months 1 and 6, allowing each trajectory to have three distinct slopes. Interaction terms of study group and time were included starting from 1-month assessment to capture differences in trajectories between assessment-only group and intervention group. Likelihood ratio tests were used to test whether random intercepts or random slopes (i.e., between-person variability around the average growth curve) are required. Leisure-time PA and transport-related PA were modelled as negative-binomial variables due to strongly right-skewed distributions. Incidence rate ratios (IRRs) were reported for both PA outcomes. Overall ST was square root transformed to account for its slightly right-skewed distribution and then modelled as a continuous variable. A maximum likelihood estimator was used. Models were estimated under a missing at random assumption using all available data from participants with responses on the outcome variable on at least one time point and with complete responses on covariates. In addition to sex and age, results were adjusted for socio-demographic, health, and context variables that were distributed differently between follow-up responders and non-responders. Thus, education was included as a covariate as multiple logistic regression analyses had revealed that lower education was predictive for dropout (*p* < .05). Data were analyzed using Stata/SE version 14.2 [23].

## Results

### Sample characteristics

There were 98 women (64%) and 55 men (36%) with a mean age of 54.5 years (standard deviation = 6.2; Table 1). At baseline, participants were physically active for 15.6 MET-hours per week during leisure time (Median; Interquartile range [IQR]: 3.3–33.1), for 13.1 MET-hours per week during transport (Median; IQR: 2.2–26.2), and spent 40 h per week sedentarily (Median; IQR: 28.5–56.0).

**Table 1:** Baseline characteristics of the study sample (*n *= 153)

		*n*	Mean (SD) or median (IQR) or n (%)
Sex	Women	153	98 (64.1%)
Age (years)		153	54.5 (SD 6.2)
Education (years)		150	
	< 10		12 (8.0%)
	= 10		102 (68.0%)
	> 10		36 (24.0%)
Employment		150	
	Full-time or part-time		103 (68.7%)
	Not regularly		16 (10.7%)
	Not employed		31 (20.7%)
Current partnership	yes	153	108 (70.6%)
Body mass index (kg/m^2^)		152	
	< 25		42 (27.6%)
	≥ 25 and < 30		58 (38.2%)
	≥ 30		52 (34.2%)
Season		153	
	winter		17 (11.1%)
	spring		127 (83.0%)
	summer		9 (5.9%)
Recruitment		153	
	General practices		56 (36.6%)
	Job centers		34 (22.2%)
	Health insurance		63 (41.2%)
Leisure-time physical activity (MET-hours/week)		122	15.6 (IQR 3.3; 33.1)
Transport-related physical activity (MET-hours/week)		131	13.1 (IQR 2.2; 26.2)
Overall sedentary time (hours/week)		138	40.0 (IQR 28.5; 56.0)

*Notes*: *n* number of subjects, *SD* standard deviation, *IQR* interquartile range, *MET* metabolic equivalent of task

### Changes between baseline and 1-month assessment

**Fig. 2:**
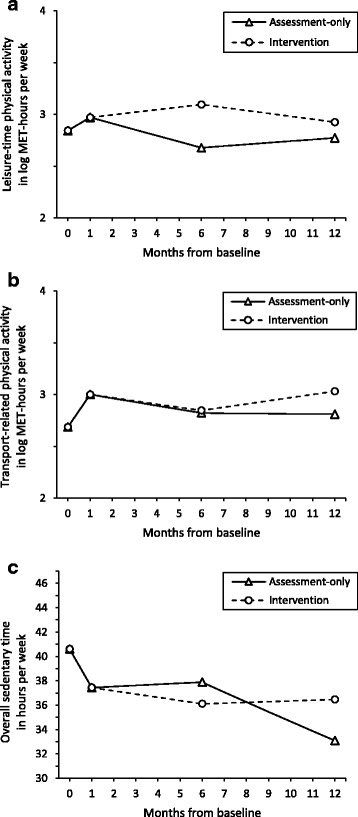
Estimated average linear growth curves for log MET-hours per week of leisure-time physical activity (**a**), log MET-hours per week of transport-related physical activity (**b**), and hours per week of overall sedentary time (**c**) separately for assessment-only group and intervention group. MET = Metabolic equivalent of task. Results were adjusted for sex, age, and education. Slope variances fixed to zero as indicated by likelihood ratio tests. To raise intuitive understanding, the outcome of sedentary time was re-calculated into hours per week

Because the intervention started after the 1-month assessment, study groups were not analyzed separately between baseline and month 1. Time spent in PA during leisure time increased over the first month by 0.13 log MET-hours per week, but the effect was not significant (IRR = 1.13, *p* = .432). Time spent in PA for transport increased significantly by 0.31 log MET-hours per week (IRR = 1.37, *p* = .023). Overall ST decreased by 1.96 square root minutes per week, but the effect was not significant (*p* = .060; Table 2, Fig. 2).

**Table 2:** Parameter estimates for latent growth models of leisure-time physical activity (*n* = 145), transport-related physical activity (*n* = 146), and overall sedentary time (*n *= 150)

	Leisure-time physical activity			Transport-related physical activity			Overall sedentary time		
	(log MET-hours/week)			(log MET-hours/week)			(sqrt min/week)		
	Est.	(SE)	*p*-value	Est.	(SE)	*p*-value	Est.	(SE)	*p*-value
Fixed effects									
Intercept	2.84	(0.16)	<.001	2.69	(0.14)	<.001	49.36	(1.19)	<.001
Slope (0 to 1 month)	0.13	(0.16)	.432	0.31	(0.14)	.023	−1.96	(1.04)	.060
Slope (1 to 6 months)	−0.06	(0.04)	.156	−0.04	(0.04)	.334	0.06	(0.28)	.842
Slope (6 to 12 months)	0.02	(0.04)	.673	−0.00	(0.03)	.951	− 0.52	(0.25)	.037
Slope (1 to 6 months × study group)	0.08	(0.05)	.111	0.00	(0.05)	.912	−0.23	(0.35)	.521
Slope (6 to 12 months × study group)	−0.04	(0.05)	.401	0.03	(0.05)	.467	0.56	(0.35)	.109
Random effects									
Intercept	0.90	(0.16)		0.85	(0.15)		7.89	(0.62)	
Slope	–			–			–		

*Notes*: *MET* metabolic equivalent of task, *Est.* estimate: mean (fixed effects), standard deviation (random effects: intercept, slope), *SE* standard error, × interaction term, − fixed at zero as indicated by likelihood ratio test

All slopes are linear. Models were adjusted for time-invariant covariates: sex, age, and education

### Changes between 1-month assessment and 12-month follow-up

#### Assessment-only group

Participants in the assessment-only group did not significantly change leisure-time PA between 1 and 6 months (IRR = 0.94, *p* = .156) or between 6 and 12 months (IRR = 1.02, *p* = .673). Transport-related PA did not significantly change between 1 and 6 months (IRR = 0.96, *p* = .334) or between 6 and 12 months (IRR = 1.00, *p* = .951). Overall ST did not change between 1 and 6 months (*p* = .842). Between 6 and 12 months, overall ST decreased significantly by 0.52 square root minutes per week (*p* = .037; Table 2, Fig. 2).

#### Intervention group

Time × study group interactions revealed that in the intervention group time trends of leisure-time PA, transport-related PA, and overall ST did not differ significantly from those in the assessment-only group both between 1 and 6 months and between 6 and 12 months (Table 2, Fig. 2).

## Discussion

There are two main findings of our study. First, participants increased self-reported transport-related PA, tended to decrease overall ST, and did not change leisure-time PA, after baseline assessment prior to the intervention period. Further, participants in the assessment-only group decreased overall ST between 6 and 12 months. These findings indicate the presence of MME. Second, in the intervention group changes over time on any of the three behaviors did not significantly differ from those observed in the assessment-only group. This indicates that the intervention did not have an effect in addition to MME.

Similar to previous findings on PA, MME was significant for one investigated PA outcome whereas comparisons on another outcome were not significant. For example, van Sluijs et al. [10] found evidence for MME on meeting recommendations on PA (30 min of at least moderate-intensity PA on at least 5 days a week), but not on other PA outcome measures, such as a categorical variable of minutes per week of moderate-to-vigorous-intensity PA. Thus, it seems to be important to consider the specific outcome measure of PA when evaluating MME.

To our knowledge, this is the first study that explicitly investigated potential effects of MME on ST. Changes during the first month after baseline were marginally significant in the expected direction indicating that MME may have altered levels of overall ST. Further, overall ST decreased after 6-month follow-up in the assessment-only group. As Ogden [24] suggested, completing a questionnaire may create new cognitions on a behavior, particularly if the behavior is novel or unfamiliar.

Starting from 1-month assessment, a random subsample of participants received a brief tailored counseling letter intervention. At 6-month follow-up, that is, shortly after the intervention period, participants in the intervention group reported an increase in leisure-time PA, whereas participants in the assessment-only group reported a reduction. Nevertheless, this difference was not statistically significant. Subsequent time trends did not indicate distinct levels of leisure-time PA between study groups after 12 months. Similar, results for transport-related PA suggest that study groups did not differ between 1 and 12 months. Whereas levels of overall ST in both groups appeared relatively constant over the course of the intervention period, time trends between 6 and 12 months suggest a less favorable development in the intervention group than in the assessment-only group. Thus, it seems that the brief tailored letter intervention did not give additional benefit over differences due to MME for all investigated outcomes. This would be consistent with the presumption of intervention effects being difficult to detect if MMEs are present in an intervention trial [8].

Three limitations of this study should be acknowledged. First, we cannot conclude which part of the research process induced MME. Baseline measurement comprised several assessments, such as self-report questionnaires on behaviors and cognitions, standardized measurement of blood pressure and waist circumference, and wearing an accelerometer. Nevertheless, previous research suggests no dose-response relationships for MME on health behaviors [13] and participants may even alter their behavior as a response to necessities like signing a consent form [8]. Second, conclusions on the presence or absence of measurement and intervention effects on any of the three behaviors should be treated with caution because our findings may suffer from a lack of power to detect differences. Third, generalizability of our findings may be compromised due to selection bias. The proportion of individuals who declined participation was high (53%) and non-participation was associated with smoking, lower education, and female sex.

Future research evaluating effects in PA and ST intervention trials should take into account that results can be biased due to MME. First, participants may change PA and ST as a reaction to baseline assessment. Therefore, an intervention may not have an effect in addition to MME. Especially in the context of brief interventions where interventions consist of short feedback letters rather than comprehensive exercise training, expected intervention effects are modest and therefore may be difficult to detect. Second, it should be considered that effects refer not alone to intervention components, but in fact to the combined impact of both intervention and assessments. Specifically in brief interventions, it should be acknowledged that assessments are part of the intervention. It may be more reasonable to compare the intervention group with controls that did not receive any assessments. Third, our findings of long-term effects of MME on ST should be verified, as it has been suggested that measurement itself could be a feasible and cost-effective public-health intervention [13].

## Conclusion

In conclusion, study results suggest the presence of measurement effects within a PA and ST intervention trial on transport-related PA and overall ST, but not on leisure-time PA. A brief tailored letter intervention did not produce effects in addition to MME. Future studies may need to consider the potential influence of MME by choosing an appropriate study design or cautious interpretation of intervention outcomes.

## Abbreviations

IPAQ: International Physical Activity Questionnaire
IQR: Interquartile range
IRR: Incidence rate ratio
MET: Metabolic equivalent of task
MME: Mere-measurement effect
PA: Physical activity
ST: Sedentary time
